# Tenacibaculum salmonis sp. nov., isolated from Atlantic salmon (Salmo salar L.) fish farmed in Chile

**DOI:** 10.1099/ijsem.0.006963

**Published:** 2025-11-14

**Authors:** Ruben Avendaño-Herrera, Rute Irgang, Pierre Lopez

**Affiliations:** 1 Universidad Andrés Bello, Laboratorio de Patología de Organismos Acuáticos y Biotecnología Acuícola, Facultad de Ciencias de la Vida, Viña del Mar, Chile; 2 Interdisciplinary Center for Aquaculture Research (INCAR), Viña del Mar, Chile; 3 Centro de Investigación Marina Quintay (CIMARQ), Universidad Andrés Bello, Quintay, Valparaíso, Chile

**Keywords:** Atlantic salmon, Chile, fish aquaculture, tenacibaculosis

## Abstract

Strain P3-BQ1^T^ is a Gram-negative, aerobic, non-spore-forming bacterium with gliding motility and filamentous cells. It was isolated in 2018 from the gills of a diseased Atlantic salmon (*Salmo salar*) during an outbreak of tenacibaculosis in a Chilean fish farm. Phylogenetic analysis based on 16S rRNA gene sequencing confirmed that strain P3-BQ1^T^ belongs to the genus *Tenacibaculum* and is most closely related to *Tenacibaculum dicentrarchi* 35/09^T^ (98.53%), ‘*Tenacibaculum pacificus*’ 18-2881-A (98.46%), *Tenacibaculum aestuariivivum* JDTF-79^T^ (98.46%), *Tenacibaculum finnmarkense* TNO010 (97.85%) and *Tenacibaculum piscium* TNO20^T^ (97.41%). The genome size of strain P3-BQ1^T^ is 2,777,603 bp, with a DNA G+C content of 29.18 mol%. The strain has an OrthoANI score lower than 95% compared to the types of all validly named *Tenacibaculum* species, with the closest neighbour being *T. dicentrarchi* 35/09^T^ (score 87.41 %). In contrast, P3-BQ1ᵀ and ‘*T. pacificus*’ 18-2881-A shared a 96.58%, thus exceeding the species threshold. This result is also supported by whole-genome *in silico* DNA–DNA hybridization/Genome-to-Genome Distance Calculator values, indicating that strains P3-BQ1^T^ and 18-2881-A are conspecific. Strain P3-BQ1^T^ contains MK-6 as its sole detectable menaquinone. The polar lipids profile includes glycolipids (*n*=2), aminolipids (*n*=3) and unidentified lipids (*n*=5). The predominant cellular fatty acids (>5 %) are C_13 : 1_, C_15 : 0_, iso-C_15 : 0_, iso-C_15 : 0_ 3-OH and summed feature 3 (C_16 : 1_ω7с/C_16 : 1_ω6с). An immersion challenge was conducted to assess the pathogenic potential in Atlantic salmon. Our findings indicate that strain P3-BQ1^T^ alone does not cause significant mortality, suggesting it is likely non-pathogenic to this species. Based on the phenotypic, phylogenetic and genotypic data presented, *Tenacibaculum salmonis* sp. nov. is proposed here with type strain P3-BQ1^T^ (=IMI 507635^T^=RGM 3581^T^), being 18-2881-A an additional strain.

**Impact Statement**

This publication proposes the 39th species within the genus *Tenacibaculum*, for which the name *Tenacibaculum salmonis* sp. nov. has been chosen to highlight the isolation source of the two strains on which the description is based. Thus, the type strain, P3-BQ1^T^, originated from diseased Atlantic salmon in Chile, and genomic analyses conducted during its characterization allowed us to conclude that the strain ‘*Tenacibaculum pacificus*’ 18-2881-A, isolated from salmonid aquaculture in Canada, is conspecific. Considering that at least eight species of this genus are pathogenic to aquatic organisms of commercial and aquaculture interest and given that strains P3-BQ1^T^ and 18-2881-A were isolated from farmed salmon affected by tenacibaculosis, we conducted an immersion challenge experiment simulating natural conditions to assess the pathogenic potential of *T. salmonis*.

## Introduction

The bacterial genus *Tenacibaculum*, belonging to the family *Flavobacteriaceae* (phylum *Bacteroidota*), comprises Gram-negative marine species. These mainly present long rods and/or filamentous cells that adhere to the surfaces of marine organisms and abiotic substrates and are also found free-living in water samples [1]. To date, 38 *Tenacibaculum* species have been described and have validly published names (https://lpsn.dsmz.de/genus/*Tenacibaculum*: accessed on 27 June 27 2025). Most *Tenacibaculum* species have been described from fish, marine sediments, macroalgae and invertebrates [1]. These are generally considered non-pathogenic, except for nine members associated with fish diseases, specifically tenacibaculosis, which causes ulcerative external lesions in commercially farmed fish worldwide [2,4]. Fish affected by this disease exhibit symptoms such as skin lesions (often ulcerative), frayed fins, tail rot, eroded mouth and haemorrhaging gills [2].

In Chile, the second largest producer of farmed Atlantic salmon (*Salmo salar*) after Norway, tenacibaculosis is the second leading cause of mortality in cultivated Atlantic salmon, accounting for 32.9% of deaths in the first half of 2023 [5]. *Tenacibaculum dicentrarchi* is primarily responsible for this disease. Since the initial report of *T. dicentrarchi* in 2016 [6], five other pathogenic species associated with salmon farming have been described, including *Tenacibaculum maritimum* [7,8], *Tenacibaculum finnmarkense* [9], *Tenacibaculum ovolyticum* [10], *Tenacibaculum piscium* [11] and, most recently, the new *Tenacibaculum bernardetii* [12].

The isolation and identification of different *Tenacibaculum* species align with the intensification of health surveillance conducted by our research team in marine fish farming cages located from the Los Lagos Region to Magallanes Region in the Chilean Patagonia (spanning almost 1,200 km from north to south). These efforts specifically focus on the detection of *T. dicentrarchi*, at farms where clinical signs of tenacibaculosis are observed. Within these surveys, various pigmented bacteria not belonging to the aforementioned species have been isolated and preserved, requiring polyphasic studies to determine taxonomic status. Herein, we report the isolation and characterization of strain P3-BQ1^T^ of the genus *Tenacibaculum* recovered from a diseased Atlantic salmon sampled in July 2018 from the Chilean Patagonia [13]. As part of the study, we also examined its relationship to another strain isolated from an external lesion of Atlantic salmon during a mouth rot outbreak in British Columbia, Canada [14].

## Isolation and maintenance

Strain P3-BQ1^T^ was isolated from the gill of a diseased Atlantic salmon weighing ~3 kg during the marine-culture phase in southern Chile (41° 28′ 9.5″ S 72° 5′ 2″ W) in July 2018. This specimen was selected from the mortality container of a cage containing 32,000 Atlantic salmon. It was chosen because it represented a fresh mortality (i.e. that day) and exhibited characteristic clinical signs of tenacibaculosis. The stocking density was 12 kg m^−^³, and the seawater temperature was 10±1 °C with a salinity of 34±1 ppt. The sampling aimed to identify the causative agent of the tenacibaculosis outbreak. The snout, gills, fins, an ulcer lesion, spleen, liver and kidney were directly streaked onto the *Flexibacter maritimus* culture media (FMM) [15]. Plates were incubated aerobically at 18 °C for 1 week and daily examined for the growth of bacterial colonies. All the experimental procedures and animal manipulations adhered to ethical guidelines for the humane treatment of live animals set forth by the Chilean National Commission of Scientific and Technological Research and were approved by the Ethics Committee for Animal Experiments of the Universidad Andrés Bello under authorization no. 015/2015.

Mixed cultures were obtained from fish samples. Yellow colonies of varying shades were selected and streaked onto fresh FMM plates to obtain pure cultures. From these cultures, a bacterial colony was picked, and DNA was extracted using the InstaGene™ Matrix (Bio-Rad Laboratories) following the manufacturer’s recommendations. This genomic DNA was then subjected to a *T. dicentrarchi*-specific PCR assay following the protocols of Avendaño-Herrera *et al*. [16], with DNA from *T. dicentrarchi* CECT 7612^T^ used as a positive control. Positive amplification with the expected 284 bp fragment was identified in colonies recovered from the snout, ulcer lesion, kidney and fin in this same sampling, while the other yellow colonies, described as P3-BQ1^T^, did not show amplification. A single colony of strain P3-BQ1^T^ was streaked onto a new FMM agar plate to ensure purification. The strain was stored in Cryobille tubes (AES Laboratories, France) with marine broth 2216 (BD Difco™) supplemented with 10% glycerol (w/v) at −80 °C until further studies.

## 16S rRNA gene sequence analysis

Colonies of strain P3-BQ1^T^ were scraped from pure culture grown on FMM agar plates, and genomic DNA extraction was performed using the InstaGene™ Matrix (Bio-Rad) following the manufacturer’s protocol. The 16S rRNA gene was amplified using the universal primer pair 27F (AGAGTTTGATCMTGGCTCAG) and 1492R (TACGGYTACCTTGTTACGACTT) [17]. The PCR reaction was carried out using GoTaq® Flexi G2 (Promega) in a final volume of 50 µl containing 10 ng of template DNA, 5 × GoTaq® Flexi Buffer (Promega), 2 mM of MgCl_2_, 10 mM of dNTPs and 200 nM of each primer. Amplification was performed on a Gene Touch TC-EA thermal cycler (Bioer Technology, Hangzhou Bioer Technology Co., Ltd.) under the following conditions: initial denaturation at 95 °C for 6 min, followed by 35 cycles of denaturation at 95 °C for 0.5 min, annealing at 52 °C for 0.5 min and extension at 72 °C for 1 min, with a final extension step at 72 °C for 10 min. Subsequently, the amplification product was sequenced by Macrogen Inc. (Santiago, Chile). The resulting 16S rRNA sequence was manually processed using the Benchling software (https://www.benchling.com/). The nearly complete 16S rRNA gene sequence, with a length of 1,366 nt (94.9% completeness), was compared to the most closely related type strains using EzBioCloud online against the 16S-based identification database (https://www.ezbiocloud.net/). Multiple alignments of all 16S rRNA sequences of *Tenacibaculum* type strains were performed using MAFFT alignment v7 (https://mafft.cbrc.jp/alignment/server/index.html; [18]), and a phylogenetic tree was reconstructed using the neighbour-joining algorithm with the Jukes–Cantor model. Node support was assessed using the bootstrap method with 1,000 replicates. Additionally, a second phylogenetic tree was constructed using the maximum likelihood method via the website https://ngphylogeny.fr [19] in ‘A la carte’ mode, i.e. the sequence alignment was performed using muscle, cleaned using the Gblocks method, and the tree was inferred using PhyML.

The nucleotide sequence showed the highest similarities to the types of various validly named *Tenacibaculum* species: 98.53% to *T. dicentrarchi* 35/09^T^ (FN545354), 98.46% to *Tenacibaculum aestuariivivum* JDTF-79^T^ (MF193601), 97.85% to *T. finnmarkense* TNO010 (GU124765), and 97.41% to *T. piscium* TNO020^T^ (GU124766). However, another notable match was found with strain ‘*Tenacibaculum pacificus*’ 18-2881-A (98.46%), which was isolated from ulcerated Atlantic salmon during the 2017–2020 mouth rot outbreak in Canada [14]. As strain 18-2881-A is not available in any bacterial culture collection, we were unable to perform further characterization analyses (e.g. physiological and chemotaxonomic studies). Nonetheless, it was included in the genomic analyses, as the corresponding data were made available through a previous publication [14].

**Fig. 1.**
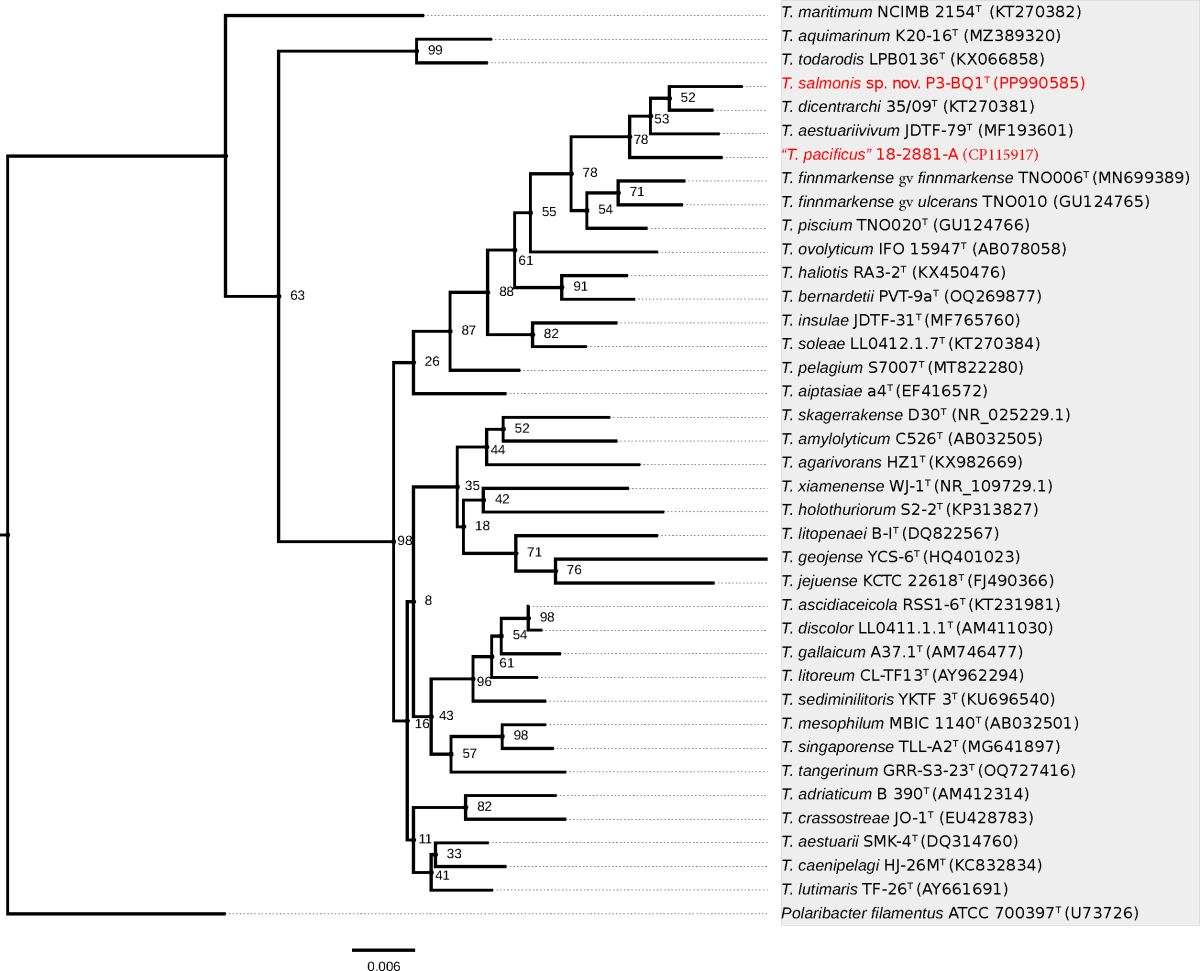
The taxonomic position of strain P3-BQ1^T^ within the phylogeny of 35 type species of the genus *Tenacibaculum*, using the neighbour-joining method with the Jukes–Cantor model based on 1,366 bp of the 16S rRNA gene. Evolutionary distances were calculated with bootstrap support from 1000 replicates. *Polaribacter filamentus* ATCC 700397^T^ was used as an outgroup.

The topology of the neighbour-joining (Fig. 1) and maximum likelihood trees (Fig. S1, available in the online Supplementary Material) exhibited considerable congruence and aligned with the results from EZBioCloud, showing that strain P3-BQ1^T^ was closely phylogenetically related to *T. dicentrarchi* 35/09^T^, its nearest neighbour species. This finding confirmed the classification of strain P3-BQ1^T^ within the genus *Tenacibaculum*.

## Whole-genome analysis, phylogeny and features

Whole-genome sequencing of strain P3-BQ1^T^ was conducted by SeqCenter (Pittsburgh, USA) using a hybrid assembly of Nanopore and Illumina reads. Assemblies were conducted using the default parameters of the Genome Assembly Service (https://www.bv-brc.org/app/Assembly2) [20]. Subsequently, the genome was deposited and annotated in the Microbial Genome Annotation and Analysis Platform [21]. The draft genome of strain P3-BQ1^T^ had a size of 2,777,603 bp, a G+C of 29.18 mol%, and consisted of 6 contigs (> 1 kb) with an N50 value of 2,703,636 bp. Gene prediction facilitated the annotation of 2,396 coding sequences, including 66 tRNA and 24 rRNA (with 8 copies of the 16S RNA). Table 1 presents comprehensive genomic characteristics of strain P3-BQ1^T^ and the respective related species.

**Table 1.** Genomic features of strain P3-BQ1^T^ and closely related species Strains: 1, P3-BQ1^T^; 2, ‘*T. pacificus*’ 18-2881-A; 3, *T. dicentrarchi* 35/09^T^; 4, *T. finnmarkense* TNO010; 5, *T. finnmarkense* TNO006^T^; 6, *T. piscium* TNO020^T^.

Characteristic	1	2	3	4	5	6
Genome status	Draft	Draft	Complete	Draft	Draft	Draft
Check M completeness (%)	99.16	97.77	98.77	99.11%	99.11	98.94
Check MK contamination (%)	0.57	0.51	0.34	0.50	0	0
Genome coverage	137 ×	99.5	363 ×	70 ×	90 ×	100 ×
Number of contig (>1 kb)	6	1	0	55	103	44
N50	2.703.636	2.791.743	2.808.268	232.000	124.200	289.600
L50	1	1	1	5	6	4
Genome size	2.780.885	2.791.743	2.808.268	2.821.318	2.933.487	2.457.154
Number of CDS	2398	2442	2355	2456	2595	2163
G+C content (mol%)	29.18	29.27	30.43	31.06	30.91	30.71
rRNA	24	27	27	0	1	1
tRNA	66	68	65	49	50	48
tmRNA	1	1	1	1	1	1
GenBank accession number	GCA_041053065.1	GCA_027941775.1	GCA_964036635.1	GCA_900239495.1	GCA_900239185.1	GCA_900239505.1

**Fig. 2.**
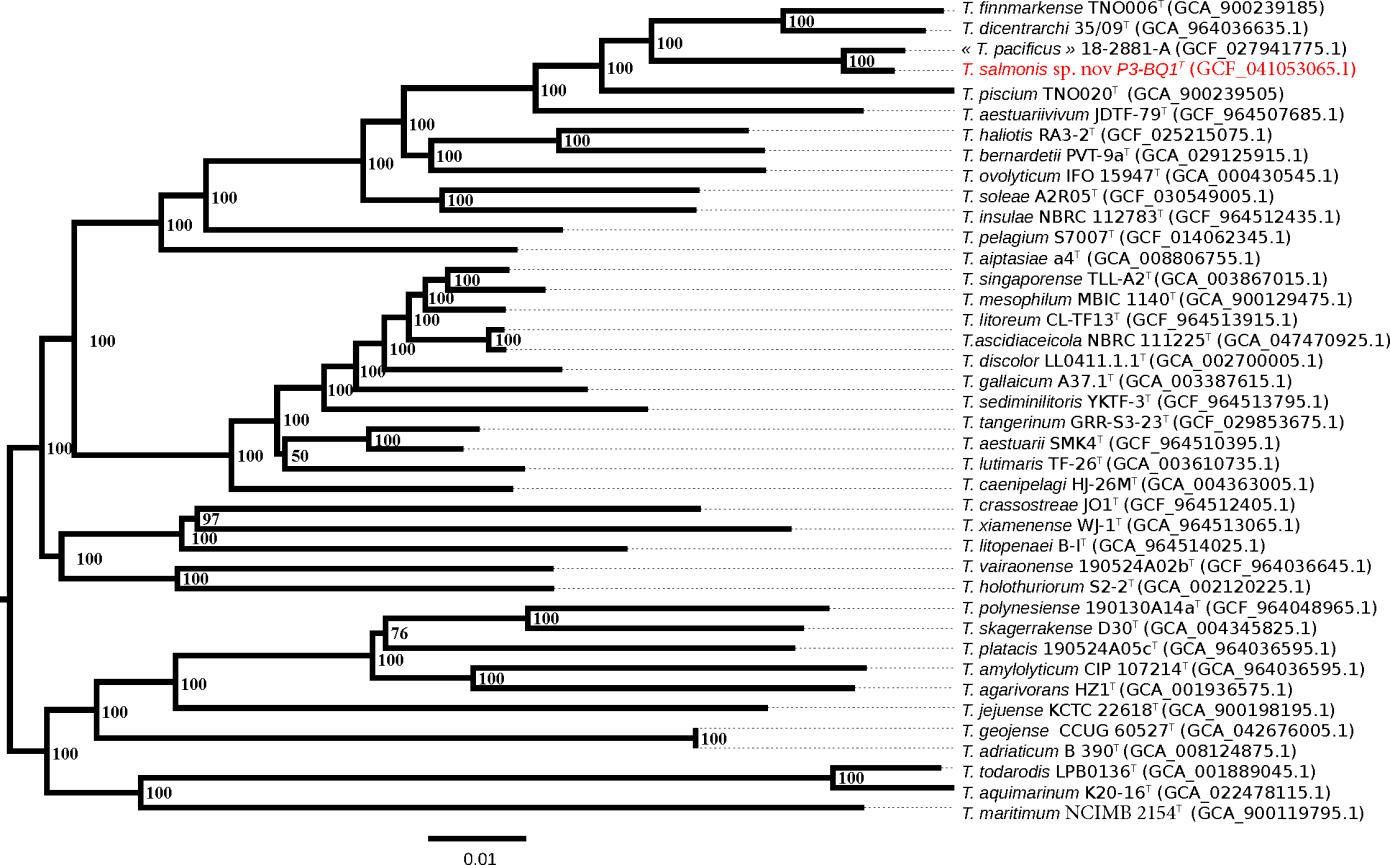
Phylogenetic tree depicting the placement of strain P3-BQ1^T^ within the *Tenacibaculum* genus, inferred from a concatenated alignment of 568 core proteome sequences (189,429 aa). The core proteome was defined as sequences sharing >80 % identity over more than 80% of their length. The tree is rooted at the midpoint. Node support values were estimated via bootstrap analysis (1,000 replicates). The JTT substitution model was used for phylogenetic inference.

 To further refine the taxonomic position of strain P3-BQ1^T^, a phylogenomic tree based on the core proteome was constructed, and evolutionary distance was calculated using the neighbour-joining method. In these analyses, the strain ‘*T. pacificus*’ 18-2881-A was included. The core proteome, which consists of proteins shared among all type strains of the genus *Tenacibaculum* with at least 80% sequence identity over 80% of the protein length, was identified. A total of 501 proteins meeting these criteria were retrieved from the Microbial Genome Annotation and Analysis Platform [21]. These protein sequences were concatenated into a single sequence for each strain (totalling 162,376 aa) using an in-house-developed R script v4.2.1 and Python scripts [22]. The alignment process involved grouping sequences into gene families, aligning them using muscle [23] implemented in the msa R package (Bioconductor) and removing any families containing incomplete orthologous sequences. Based on this alignment, a phylogenetic tree was constructed using the neighbour-joining method. The robustness of the phylogenetic tree was assessed by performing 1,000 bootstrap replicates to ensure the reliability of the inferred evolutionary relationships. The tree was rooted at the midpoint. The phylogenetic analysis confirmed the position of the P3-BQ1^T^ strain within the cluster comprising the pathogenic species *T. piscium* TNO020^T^, *T. dicentrarchi* 35/09^T^ and *T. finnmarkense* TNO006^T^, consistent with results of the 16S rRNA gene sequence-based analyses (Fig. 2).

To estimate the degree of pairwise relatedness between P3-BQ1^T^ and the closest reference strains, pairwise average nucleotide identity values were computed using OrthoANI [24] (https://www.ezbiocloud.net/tools/orthoani), while digital DNA–DNA hybridization values were evaluated using the Genome-to-Genome Distance Calculator v3.0 and Formula: 2 (http://ggdc.dsmz.de/ggdc.php) [25]. The OrthoANI values range was from 74.69% (strain HZ1^T^ of *T. agarivorans*) to 87.41 % (strain 35/09^T^ of *T. dicentrarchi*) when compared with P3-BQ1^T^, which is below the 95% cut-off for bacterial species boundaries [26]. In contrast, between strains P3-BQ1^T^ and 18-2881-A, the value was above the threshold, that is, 96.58%. The lowest genome-to-genome distance for strain P3-BQ1^T^ was with *Tenacibaculum* strain 35/09^T^, at 12.46 [DNA–DNA hybridization estimate (general lineal model-based): 33.50% (31.1–36 %)], which is below the 70 % cut-off above which isolates are considered conspecific [27]. However, DNA–DNA hybridization analysis further indicated that the strain 18-2881-A likely belongs to the same species as P3-BQ1^T^. In fact, the DNA–DNA hybridization estimate (general lineal model-based) was 69.90% (66.9–72.8 %). Although the DNA–DNA hybridization estimate value between the two strains is around the threshold level, when combined with the OrthoANI value, it supports the conclusion that strains P3-BQ1^T^ and 18-2881-A belong to the same species [28]. On the other hand, a possible clonal relationship between the two bacteria can be ruled out, as the values would be expected to be much higher. In fact, this leads to the hypothesis that they may represent evolutionary lineages in the process of speciation.

## Genomic insights into secretion systems and virulence factors

As members of the genus *Tenacibaculum*, the presence of the type IX secretion system (T9SS) was investigated in strains P3-BQ1^T^ and 18-2881-A. For this purpose, T9GPred, designed to identify genes associated with T9SS, was used (https://cb.imsc.res.in/t9gpred/prediction/T9SSpred) [29]. The analysis indicated the presence of an efficient T9SS, the details of which are summarized in Table S1. Proteins secreted by T9SS have been found to possess either a TIGR04183 (type A) or TIGR04181 (type B) carboxyl-terminal domain [30]. The genome was searched for all predicted proteins containing one of these two domains. Twenty-four carboxyl-terminal domain docking sites were identified that contained one of these two domains (Table S1). Among these, three were described as putative peptidases (family M43 or S8), and five were likely involved in cell recognition/adhesion, characterized by domains such as Ig, VWF, laminin G, cadherin or SprB adhesin. Additionally, an examination was conducted to determine if the predicted proteins were present in the genomes of other closely related pathogenic *Tenacibaculum* species, i.e. known fish pathogens within the same cluster, such as *T. dicentrarchi*, *T. finnmarkense* and *T. piscium*. This was done through blasting analysis of the respective protein sequences. Interestingly, between 10 and 14 of these carboxyl-terminal domains docking sites were also found in *T. finnmarkense*, *T. piscium*, *T. ovolyticum* or *T. dicentrarchi* (Table S2).

## Physiology and chemotaxonomy

Strain P3-BQ1^T^ was subjected to characterization by morphological, phenotypic and biochemical tests following the protocols described by Olsen *et al*. [31], Piñeiro-Vidal *et al*. [32] and Suzuki *et al*. [33], as recommended for the description of the species most closely related to P3-BQ1^T^ within the genus *Tenacibaculum*. Additionally, *T. dicentrarchi* CECT 7612^T^, the closest phylogenetic type strain, was obtained from the Spanish Type Culture Collection (CECT) (Spain) and included in each phenotypic and biochemical assay for comparative purposes. Two other type strains of the genus *Tenacibaculum*, *T. finnmarkense* TNO006ᵀ and *T. piscium* TNO020ᵀ, were included in the biochemical comparison (Table 2). The results for these type strains were obtained from the literature [9,11].

**Table 2.** Differential characteristics between strain P3-BQ1^T^ and the most closely related *Tenacibaculum* species Strains: 1, P3-BQ1^T^; 2, *T. dicentrarchi* CECT 7612^T^ (this study); 3, *T. finnmarkense* TNO006^T^ [31]; 4, *T. piscium* TNO020^T^ [31].

Characteristic	1	2	3	4
Source of strain	Atlantic salmon Gill	Disease sea bass Skin	Atlantic salmon Skin ulcer	Atlantic salmon Skin ulcer
Year of isolation	2018	< 2009	2011	1998
Country	Chile	Spain	Norway	Norway
Catalase/oxidase	– / +	+ / +	+ / +	w / w
Congo red	w	+	w	w
Growth at/with				
pH range (optimum)	6.0–9.0(7.0)	6.0–9.0 (6.0–9.0)	4.0–10.0	4.0–10.0
Seawater (%)	50–100 (70–100)	50–100 (70–100)	10–100 (50–100)	10–100 (20–100)
Temperature range (°C)	5–22 (5–20)	5–22 (5–20)	4–22 (15)	4–25 (15–22)
Hydrolysis of				
Casein	–	–	+	–
l-Tyrosine	–	–	+	–
Tween 80	+	+	w	–
Carbon sources				
Glutamate	+	w	–	–
l-Proline	–	–	–	w
Ribose	–	–	–	w
Sucrose	–	–	–	w
Nitrate reduction	+	–	– or +	–
Enzymatic activity				
Esterase (C4)	–*, –^†^	–*, +^†^	+^†^	+^†^
Esterase lipase (C8)	w, –	+, +	+	–
Lipase (C14)	–, –	–, –	w	–
Cystine arylamidase	–, +	w, +	+	+
Trypsin	–, +	–, –	w	+
G+C (mol%)	29.18	31.3	30.91	30.71

Results presented for enzymatic activity: *prepared with saline solution.

†sterilized seawater.

−, negative; +, Positive; w, weakly positive.

Strain P3-BQ1^T^ was routinely grown on FMM and marine 2216 agar plates, incubated at 18 °C for 48–72 h. Colonies were circular and raised slightly (either convex to flat, 3–4 mm in diameter) on marine 2216 agar and with spreading edges on FMM agar. Growth was also assessed on various media, including defibrillated sheep blood agar plates (5 % v/v), FMM, marine 2216 agar and Columbia base agar (Oxoid) supplemented with 1.5% NaCl (w/v). Growth was only observed on the FMM and marine 2216 agar plates, but with no haemolysis detected.

Gram staining and cell morphology were examined using light microscopy at ×1,000 magnification with a Motic BA410 Elite microscope. Additionally, cell morphology was analysed using scanning electron microscopy at the external microscopy facility of the University of Concepción. To assess gliding motility, 20 µl of undiluted cell culture was placed on microscope cover slips and observed under ×1,000 magnification. Gram-negative, rod-shaped cells (Fig. S2) exhibiting gliding motility were observed in 24 h cultures in Marine 2216 broth. Catalase and oxidase activities were determined by flooding cells with 3% hydrogen peroxide (v/v) (Merck Millipore) and the oxidase reagent dropper (BD Difco), respectively. Strain P3-BQ1^T^ tested negative for catalase but positive for oxidase activity. The presence of flexirubin and Congo red was assessed following the methods described by Bernardet *et al*. [34], with absorption observed only for Congo red. Growth was investigated by seeding the strain on marine 2216 agar and FMM agar plates at 5, 10, 15, 18, 20, 22, 25 and 37 °C. Optimal growth occurred between 10 and 18 °C, with growth observed from 5 to 22 °C. No growth was observed at 25 °C or higher after 21 days of incubation, regardless of the culture media used.

For comparative purposes, results were contrasted against those reported for the closest related species of *Tenacibaculum* (Table 2). pH and salinity tolerance were tested at 18 °C and 22 °C. Tolerance to different pH ranges was assessed using marine 2216 broth, with values ranging from 4 to 10 (adjusted in 1.0-unit increments using 1 M NaOH and 1 M HCl buffers). pH levels were verified after sterilization. Growth was observed between pH 6.0 and 9.0, with optimal growth occurring at pH 7.0. Tolerance to various NaCl concentrations (w/v) was tested using marine 2216 broth supplemented with 0–6.0 % NaCl (w/v) (1% increments). Optimal growth was observed without NaCl supplementation, and no growth was observed at higher NaCl concentrations, regardless of the temperature tested. Growth in different percentages of seawater was tested using FMM broth [30]: 0, 10, 20, 30, 50, 70 and 100%. Optimal growth was observed only in 50–100 % seawater, regardless of the temperature tested. Hydrolysis of 0.4% starch (w/v) (Winkler) and 0.4% l-tyrosine (w/v) (Sigma-Aldrich), 2% gelatine (w/v) (BD Difco™) and 1% Tween 80 (v/v) (AppliChem GmbH) was tested using FMM agar as the basal medium. Strain P3-BQ1^T^ exhibited hydrolytic activity only towards gelatine and Tween 80. Utilization of different carbon sources was tested according to the methods of Olsen *et al*. [31] and Suzuki *et al*. [33]. The basal medium consisted of 0.2 g NaNO_3_, 0.2 g NH_4_Cl, 0.05 g yeast extracts, 15 g bacteriological agar (BD Difco™) and 36 g Sea Salts (Sigma Aldrich Co.) supplemented with d-glucose, d-galactose, d-ribose, l-glutamate and sucrose at 0.4 % (w/v) each. The isolate grew only on plates supplemented with glutamate. Nitrate reduction was investigated according to Piñeiro-Vidal *et al*. [32], confirming positive nitrate reduction by the strain. Enzyme activities were determined using API^®^ZYM (bioMérieux, France), and two suspension media were tested: a saline solution [35] and sterilized seawater [31]. Strips were incubated at 20 °C for 48 h. The strain exhibited positive and strong enzymatic activities for acid phosphatase, alkaline phosphatase, leucine arylamidase, valine arylamidase and naphtol-AS-BI-phosphohydrolase, regardless of the resuspension solution. Enzymatic activities for cystine arylamidase and trypsin were found to be positive when seawater was used. However, negative results were observed for esterase (C4), esterase lipase (C8), lipase (C14), *α*-chemotrypsin, *α*- and *β*-galactosidase, *β*-glucuronidase, *α*- and *β*-glucosidase, *N*-acetyl-*β*-glucosaminidase, *α*-mannosidase and *α*-fucosidase, regardless of whether the saline solution or seawater was used as the suspension medium. Table 2 outlines the primary phenotypical distinctions observed among the closest related species.

Polar lipid and respiratory quinone analyses of strain P3-BQ1^T^ were conducted by DSMZ Services, Leibniz-Institut DSMZ-Deutsche Sammlung von Mikroorganismen und Zellkulturen GmbH (Braunschweig, Germany). The strain was cultured on marine 2216 agar plates and incubated at 18 °C for 48 h. After harvesting, cells were suspended in an isopropanol/water solution (1:1, v/v) and analysed according to Bligh and Dyer [36] and Tindall *et al*. [37]. The polar lipids identified included glycolipids (*n*=2), aminolipids (*n*=3) and unidentified lipids (*n*=5) (Fig. S3). The respiratory quinone system consisted solely of MK-6 (100%).

The cellular fatty acid composition of strain P3-BQ1^T^ was determined by DMSZ (Germany) using Fatty Acid Methyl Esters (FAMEs) [38] and gas chromatography in an Agilent GC-MS 7000D system [39]. The fatty acid profile of *T. dicentrarchi* CECT 7612^T^, the closest phylogenetic species, was also analysed for comparative purposes by requesting the service from CECT. Peaks were identified based on retention time and mass spectra. The major fatty acids (>5 %) identified in strain P3-BQ1^T^ were C_13 : 1_, C_15 : 0_, iso-C_15 : 0_, iso-C_15 : 0_ 3-OH, iso-C_15 : 0_, C_17 : 1_ ω6c and summed feature 3 (C_16 : 1_ω7с/C_16 : 1_ω6с) (Table S3).

## Virulence potential of strain P3-BQ1^T^ in fish

The virulence potential of strain P3-BQ1^T^ was determined through immersion-based infection trials involving 35 Atlantic salmon (~300 g) with no prior disease histories, following challenge methodologies previously proposed for other pathogenic *Tenacibaculum* species [6,11]. Before the challenge, the fish were screened to confirm they were free from endemic pathogens (e.g. infectious pancreatic necrosis virus, infectious salmon anaemia virus, *Piscirickettsia salmonis*, *Renibacterium salmoninarum*, *T. maritimum*, *T. dicentrarchi* and other *Tenacibaculum* spp.). For this purpose, five fish were euthanized, and samples of kidney, liver, spleen, gill and muscle were collected and submitted to a private diagnostic laboratory accredited by the National Fisheries and Aquaculture Service of Chile for pathogen certification. For the trial, fish were randomly assigned to three groups of 10 individuals each: (1) inoculated with strain P3-BQ1^T^, (2) FMM medium only and (3) a control group (no bacterial inoculation). Each group was housed in 100-l tanks filled with aerated seawater (36‰ salinity, maintained at 13 ± 0.5 °C). Prior to the experimental challenge, all fish underwent a 20-day acclimation period.

The inoculum of strain P3-BQ1^T^ was grown in FMM broth for 48 h at 18 °C with agitation at 100 r.p.m. Fish were exposed to the bacteria via immersion for 120 min in aerated seawater containing a bacterial concentration of 1.54×10^6^ c.f.u. ml^−1^, as quantified by direct plate counting. In parallel, another group of fish was exposed to an FMM medium bath under the same conditions and duration, while the control group was left unmanipulated. After 2 h of exposure, fish from each group were redistributed into 80-l tanks at a density of five fish per tank, with the challenge performed in duplicate. Fish were fed daily at 1.5% of their body weight, and tank water was renewed every other day to eliminate faecal matter and nitrogenous waste. Fish were observed daily over 21 days for behavioural changes and/or clinical signs. During the challenge period, up to three dead fish were sampled per day per treatment. Dead fish were removed daily and examined for clinical signs of tenacibaculosis. To confirm if the inoculated bacterium caused death, attempts were made to re-isolate the bacteria from external (e.g. gills, skin lesions and ulcers) and internal (e.g. kidney, liver and spleen) samples. These samples were streaked onto FMM agar plates and incubated aerobically at 18 °C for 1 week. Phenotypic tests were conducted to identify colonies of the suspected P3-BQ1ᵀ strain.

**Fig. 3.**
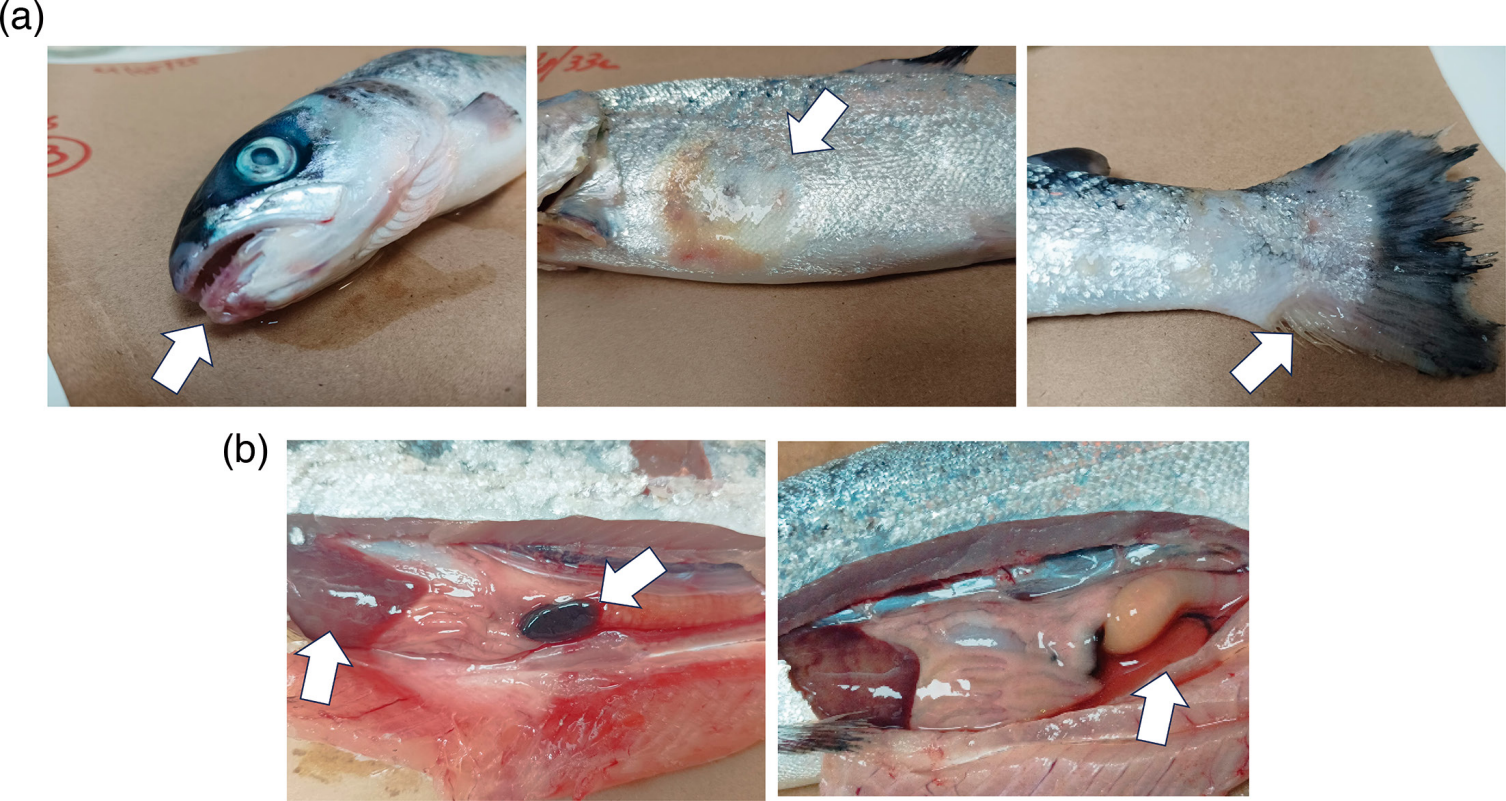
Mortality of Atlantic salmon specimens challenged by immersion with strain P3-BQ1ᵀ, showing external skin lesions, tail damage and mouth erosion (**a**) and internal signs of pale liver and enlarged spleen with ascitic fluid (**b**).

Of the ten Atlantic salmon exposed to strain P3-BQ1ᵀ via immersion, only two fish died. These individuals exhibited scale loss, skin lesions, haemorrhages on the fins, snout and operculum, as well as petechiae on the abdomen and frayed fins (Fig. 3). Some of these signs are consistent with those caused by tenacibaculosis-inducing agents [3]. Internally, both fish showed haemorrhaging, pale livers and enlarged spleens. A similar number of mortalities was recorded in the group treated with FMM medium only; however, no external or internal lesions or clinical signs were observed in these fish, which could be attributed to stress caused by handling and exposure to the FMM medium during the bath. As expected, no mortality was observed in the negative control groups during the experiment.

In the FMM plates from the treatment with strain P3-BQ1ᵀ, mixed colonies were observed, and microscopic examination revealed the presence of Gram-negative bacteria with a filamentous morphology compatible with *Tenacibaculum* in only one of the colonies isolated from gill tissue. However, it was not possible to purify the colony for biochemical analyses. The remaining mixed cultures displayed colony and bacterial morphotypes that were markedly different from those typical of strain P3-BQ1ᵀ. Avendaño-Herrera *et al*. [6] justify the presence of mixed cultures in the gills because this tissue is in contact with the seawater environment of the tanks. In fact, differentiating *Tenacibaculum* species on culture media remains challenging [40]. Moreover, the isolation of pure cultures from internal organs in cases of tenacibaculosis is very difficult in salmonid species [13].

Given the absence of diagnostic methods based on PCR or similar techniques, we were unable to identify the bacterium in some tissues of the deceased fish. Further studies are required to fulfil Koch’s postulates and to confirm or rule out that the bacterium P3-BQ1^T^ is a new causative agent of tenacibaculosis in farmed salmonids.

## Taxonomic conclusions

In conclusion, we propose that strain P3-BQ1ᵀ represents a novel bacterial species, designated *T. salmonis*, based on 16S rRNA gene sequence analysis, DNA–DNA hybridization and OrthoANI comparisons, including phylogenies derived from both 16S rRNA gene sequences and whole-genome analyses. Our molecular analyses demonstrate that the type strain is related at the species level to the Canadian strain 18-2881-A. Biochemical profiling using classical tube and plate methods, as well as commercial API systems, revealed distinctive phenotypic characteristics of this new species.

## Description of *Tenacibaculum salmonis* sp. nov.

*Tenacibaculum salmonis* (sal.mo’nis. L. gen. n. *salmonis*, of salmon, since it was isolated from Atlantic salmon).

Cells are Gram-stain-negative, rod-shaped and exhibit gliding motility. Degenerative spherical cells become abundant after 8 days of culturing. Colonies on marine 2216 agar are circular, raised to flat, smooth and non-adherent to agar. They exhibit an orange/brownish colour and reach up to 5 mm in diameter after 72 h of incubation at 18 °C. Colonies on FMM agar plates share these characteristics but appear pale yellow with spreading edges. Growth is also observed on FMM and marine 2216 agar plates supplemented with sheep blood, although no hydrolysis is detected. The strain is catalase-negative and oxidase-positive, negative for flexirubin and positive for Congo red. Growth occurs at 5–22 °C (optimal at 10–18 °C) but not at 25 °C, regardless of the culture media used. Salinity tolerance is observed in marine 2216 broth without added NaCl and in FMM prepared with 50–100% seawater. The strain hydrolyses gelatine and Tween 80 but not casein or l-tyrosine. It shows weak growth on agar plates prepared with l-glutamate, but no growth is observed on plates with d-galactose, d-glucose, ribose or sucrose. Nitrate is reduced to nitrite. In the API^®^ZYM, reactions are strongly positive for acid phosphatase, alkaline phosphatase, leucine arylamidase, valine arylamidase and naphtol-AS-BI-phosphohydrolase. Enzymatic activities are positive for cystine arylamidase and trypsin when seawater is employed. All remaining reactions are negative, regardless of the suspension medium used. The major fatty acids (>5 %) identified are C_13 : 1_, C_15 : 0_, C_15 : 1_, iso-C_15 : 0_, iso-C_15 : 0_ 3-OH, anteiso-C_15 : 0_, C_17 : 1_ ω6 and summed feature 3 (C_16 : 1_ω7с/C_16 : 1_ω6с). Polar lipids include glycolipids (*n*=2), amonilipids (*n*=3) and unidentified lipids (*n*=5). The predominant menaquinone detected is exclusively MK-6 (100%).

The type strain, P3-BQ1^T^ (=IMI 507635^T^=RGM 3581^T^) was isolated from the gill of an Atlantic salmon farmed in Chile. The GenBank accession number for the 16S rRNA of strain P3-BQ1^T^ is PP990585, and the genome assembly has been deposited under accession JBFCVV000000000. The DNA G+C content is 29.18%.

## Supplementary material

10.1099/ijsem.0.006963 Uncited Supplementary Material 1.

10.1099/ijsem.0.006963 Uncited Table S1.

10.1099/ijsem.0.006963 Uncited Table S2.

## Abbreviations

CECT: Spanish Type Culture Collection
FAME: Fatty Acid Methyl Ester
FMM: *Flexibacter maritimus* culture media
T9SS: type IX secretion system
