# Supplementary material for: Tenacibaculum salmonis sp. nov., isolated from Atlantic salmon (Salmo salar L.) fish farmed in Chile
Source: Int J Syst Evol Microbiol. 2025 Nov 14;75(11):006963. doi: 10.1099/ijsem.0.006963 (PMC12617846; doi:10.1099/ijsem.0.006963)
Supplement: Uncited Supplementary Material 1. [file ijsem-75-06963-s001.pdf]

Tenacibaculum salmonis sp. nov., isolated from Atlantic salmon (*Salmo salar* L.) fish farmed in Chile

1.1. Authors name

Ruben Avendaño-Herrera[1,2,3]*, Rute Irgang[1,2], Pierre Lopez[1,2]

1.2. Affiliations

[1]Universidad Andrés Bello, Laboratorio de Patología de Organismos Acuáticos y Biotecnología Acuícola, Facultad de Ciencias de la Vida, Viña del Mar, Chile.

[2]Interdisciplinary Center for Aquaculture Research (INCAR), Viña del Mar, Chile.

[3]Centro de Investigación Marina Quintay (CIMARQ), Universidad Andrés Bello, Quintay, Valparaíso, Chile.

1.3. Corresponding author

R. Avendaño-Herrera. Universidad Andrés Bello, Quillota 980, Piso 4-Torre C, Viña del Mar, Chile. Tel.: +56 968989187; E-mail: reavendano@yahoo.com or ravendano@unab.cl

Supplementary Figure

Figure S1 The taxonomic position of *Tenacibaculum salmonis* P3-BQ1^T among the 35 type species of the genus *Tenacibaculum*. *Polaribacter filamentus* ATCC 700397^T was used as an external group.

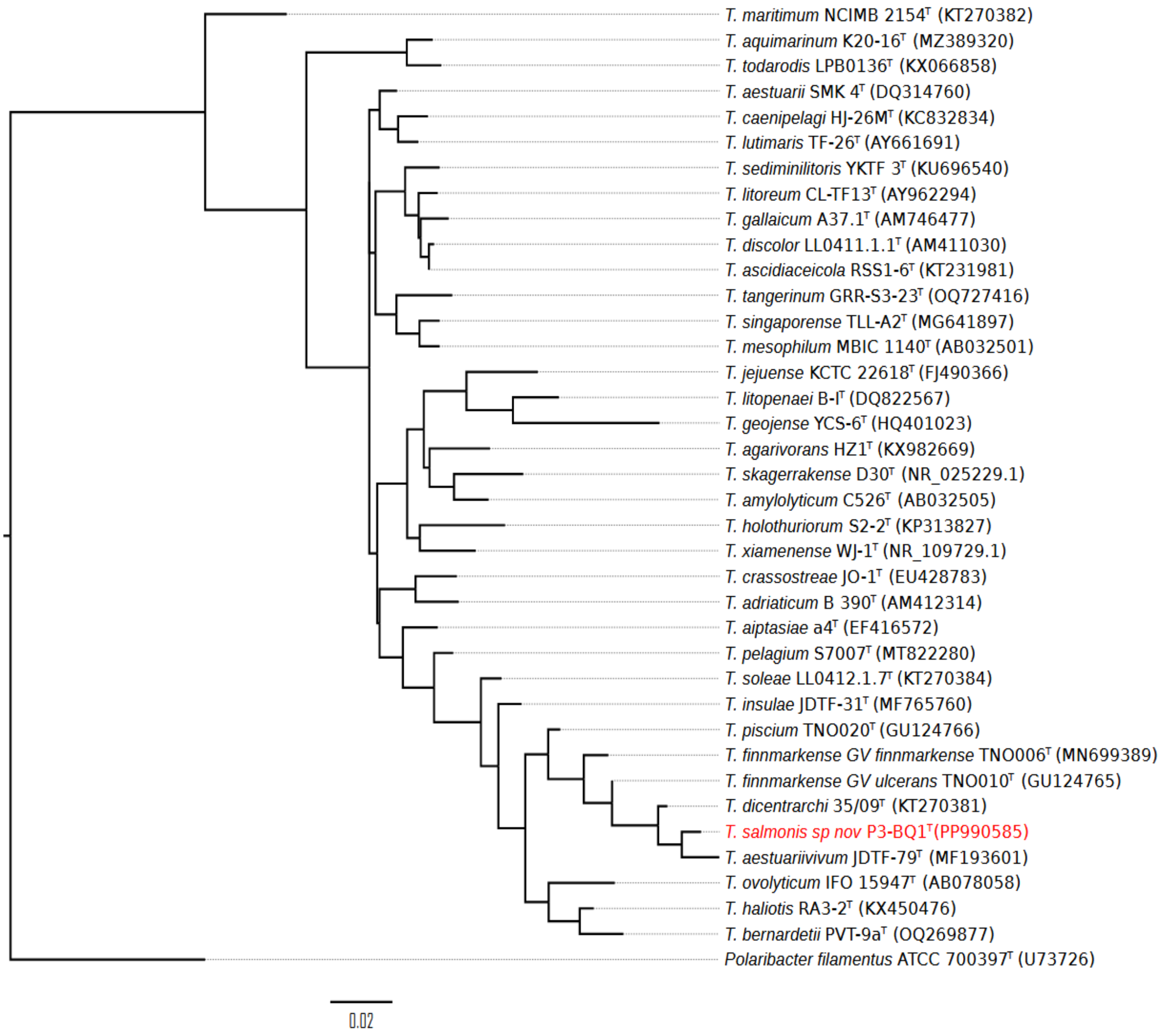

Figure S2 Scanning Electron Microscopy (SEM) of strain P3-BQ1^T after 48 h of incubation at 18ºC. Bar = 1 µm.

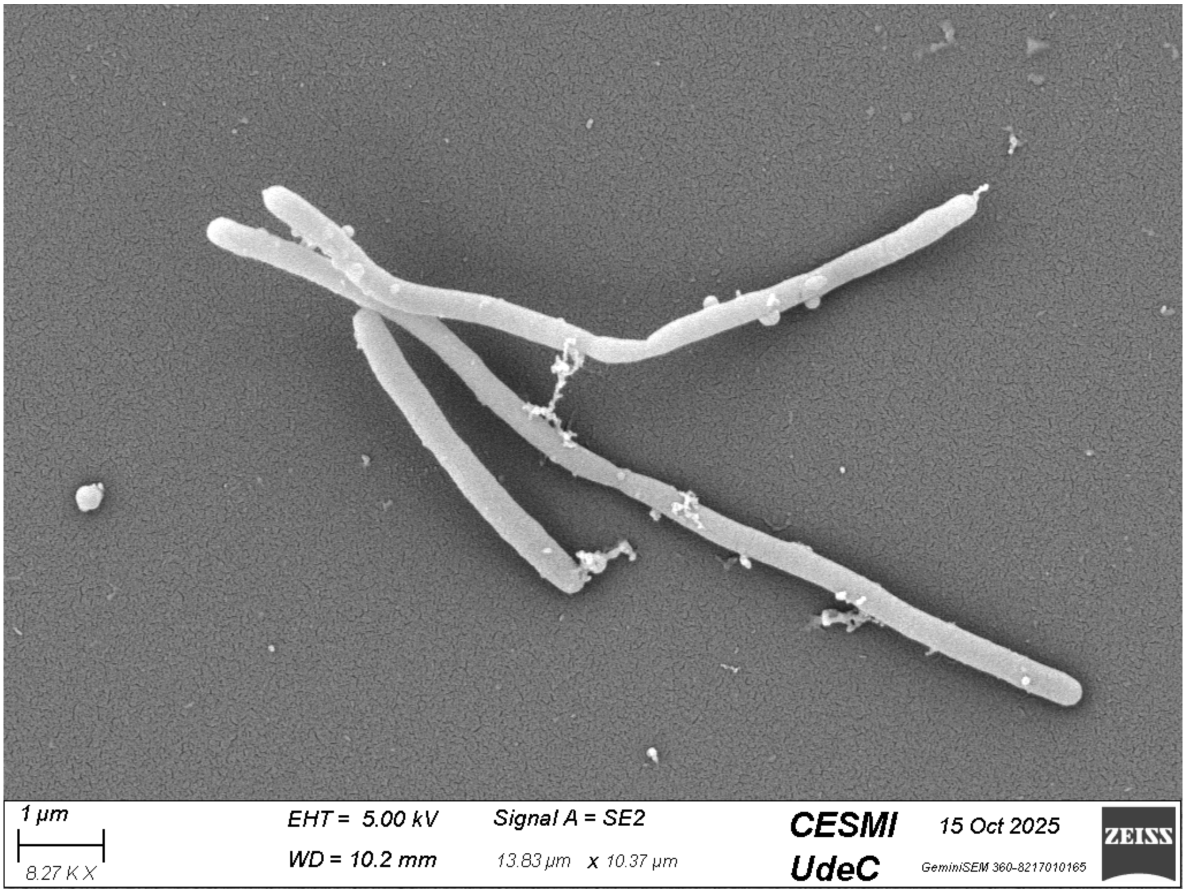

Figure S3 Polar lipids profile of *Tenacibaculum salmonis* P3-BQ1^{T} separated by two-dimensional silica gel thin layer chromatography. AL, aminolipid; GL, Glycolipid; and L, lipids.

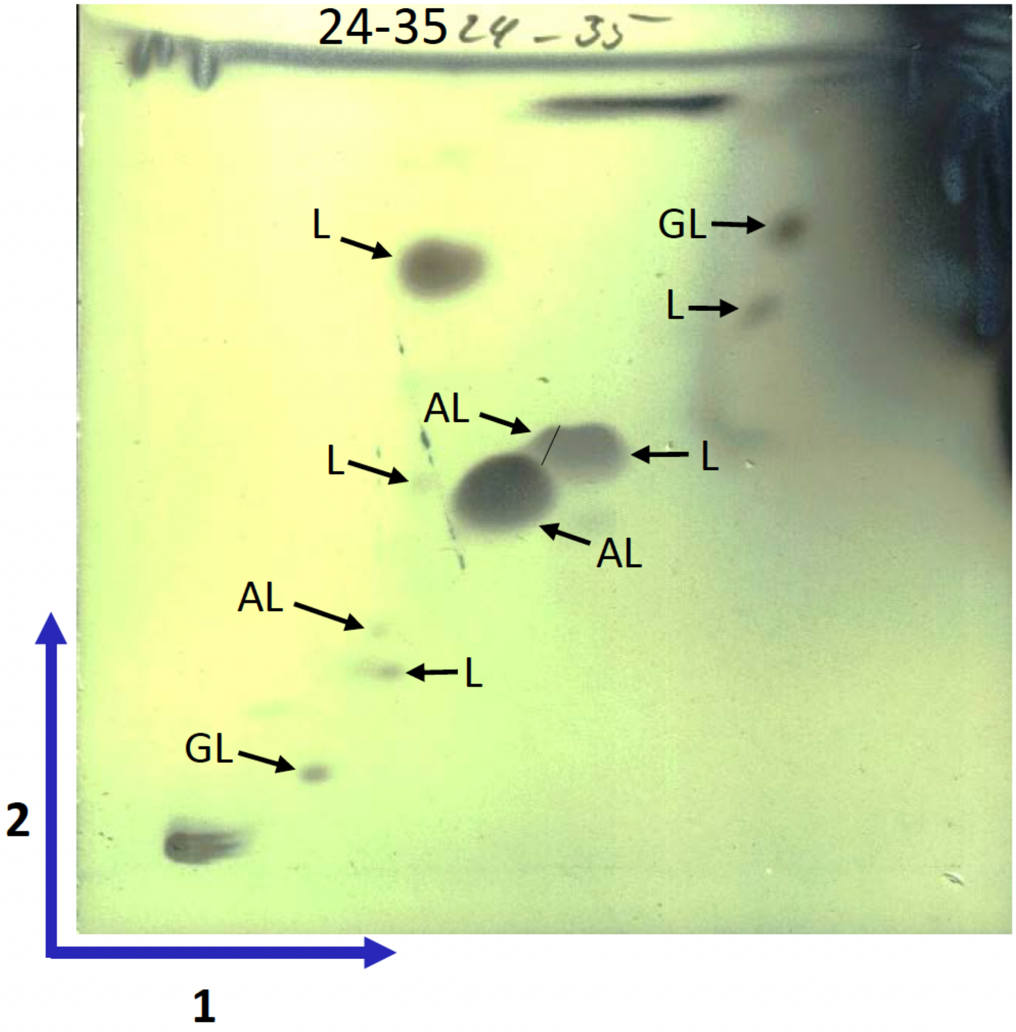

Table S3. Cellular fatty acid composition of strain P3-BQ1^T and the most closely related *Tenacibaculum* species according to whole-genome analysis. Strains: 1, P3-BQ1^T; 2, *T. dicentrarchi* CECT 7612^T (this study); 3, *T. finnmarkense* TNO006^T [31]; and 4, *T. piscium* TNO020^T [31]. –, not detected and TR, traces (< 1.0%). Major fatty acids (>5%) are shown in bold.

**Fatty acid**	**1**	**2**	**3**	**4**
Straight chain:				
$C_{12:0}$	TR	–	–	–
$C_{12:1}$ at 11-12	–	TR	–	–
$C_{13:0}$	TR	TR	–	–
$C_{13:1}$ at 12-13	**6.3**	1.2	–	–
$C_{14:0}$	3.6	1.4	2.2	1.6
$C_{15:0}$	**5.8**	–	**6.4**	2.2
$C_{16:0}$	2.6	1.3	–	–
Branched:				
iso-$C_{12:0}$	0.5	–	–	–
iso-$C_{13:0}$	0.8	1.2	1.3	0.5
iso-$C_{14:0}$	3.2	2.6	2.2	2.4
iso-$C_{15:0}$	**13.0**	**16.8**	**11.5**	**11.1**
iso-$C_{15:1}$	–	**7.7**	**8.2**	**9.6**
iso-$C_{16:0}$	0.8	0.9	0.4	1.4
iso-$C_{16:1}$	0.7	2.3	TR	3.3
anteiso-$C_{13:0}$	TR	TR	–	–
anteiso-$C_{15:0}$	**7.0**	**12.3**	**13.9**	**14.0**
anteiso-$C_{15:1}$	0.4	1.0	1.3	2.1
Unsaturated:				
$C_{14:1}\omega 5c$	TR	TR	–	–
$C_{14:1}\omega 6c$	TR	–	–	–
$C_{14:1}\omega 7c$	1.0	–	–	–
$C_{14:1}\omega 10c$	1.0	–	–	–
$C_{15:1}\omega 6c$	2.9	4.3	**6.4**	3.2
$C_{15:1}\omega 11c$	0.9	–	–	–
$C_{16:1}\omega 5c$	2.3	1.7	2.0	2.8
$C_{17:1}\omega 6c$	**5.3**	2.5	4.4	2.4
$C_{17:1}\omega 8c$	TR	TR	–	–
$C_{18:1}\omega 5c$	–	0.4	–	–
iso-$C_{15:1}\omega 10c$	**7.0**	**7.7**	**8.2**	–
iso-$C_{14:1}$ $\omega 6c$	TR	–	–	–
Hydroxy:				
$C_{15:0}$ 2-OH	0.3	0.9	1.2	0.7

$C_{17:0}$ 2-OH	–	0.6	–	–
$C_{10:0}$ 3-OH	–	TR	–	–
$C_{14:0}$ 3-OH	0.7	–	–	–
$C_{15:0}$ 3-OH	**-**	2.3	2.6	TR
$C_{16:0}$ 3-OH	3.3	3.8	**7.0**	4.4
iso-$C_{15:0}$ 3-OH	**5.7**	**12.3**	**11.1**	**8.2**
iso-$C_{16:0}$ 3-OH	1.2	4.4	2.3	4.0
iso-$C_{17:0}$ 3-OH	0.9	2.5	1.0	1.6
$C_{18:0}$ 10-methyl	–	TR	–	–
Summed feature:				
3[†]	**20.8**[†]	**12.9**	**9.7**	**17.7**
4[§]	1.3	–	–	–
9	0.3	0.8	–	–

[†]Summed feature 3 comprised $C_{16:1}\omega7c/C_{16:1}\omega6c$ and/or iso-$C_{15:0}$ 2-OH; [§], Summed feature 4 comprised iso-$C_{17:1}\omega5c$ and/or $C_{15:0}$ 3-OH, and summed feature 9 comprised iso-$C_{17:1}\omega9c$ and/or $C_{16:0}$ 10-methyl
